# A Circularly Polarized Non-Resonant Slotted Waveguide Antenna Array for Wide-Angle Scanning

**DOI:** 10.3390/s24103056

**Published:** 2024-05-11

**Authors:** Guodong Han, Weihang Liu

**Affiliations:** The 54th Research Institute of China Electronics Technology Group Corporation, Shijiazhuang 050081, China; liu_weihang@126.com

**Keywords:** slotted waveguide array, circular polarization, wide-angle scanning, non-resonant array, phased array antenna, radar sensor

## Abstract

A compact circularly polarized non-resonant slotted waveguide antenna array is proposed with the aim of achieving wide-angle scanning, circular polarization, and low side-lobe levels. The designed antenna demonstrates a scanning range of +11° to +13° in the frequency domain and a beam scanning range of −45° to +45° in the phase domain. This design exhibits significant advantages for low-cost two-dimensional electronic scanning circularly polarized arrays. It employs a compact element that reduces the aperture area by 50% compared to traditional circular polarization cavities. Additionally, the staggered array method is employed to achieve an element spacing of 0.57λ within the azimuth plane. Isolation gaps were introduced into the array to enhance the circular polarization performance of non-resonant arrays. The Taylor synthesis method was employed to reduce the side-lobe levels. A prototype was designed, fabricated, and measured. The results indicate superior radiation efficiency, favorable VSWR levels, and an axis ratio maintenance below 3 dB across the scanning range. The proposed antenna and methodology effectively broaden the beam scanning angle of circularly polarized slotted waveguide array antennas.

## 1. Introduction

The slotted waveguide antenna offers notable advantages, such as a high power capacity, low loss, and low side-lobe levels; thus, it is extensively utilized in radar applications. A circularly-polarized antenna possesses the capability to receive multiple polarized waves, and the circularly polarized wave has smaller energy losses when penetrating rain or fog areas [1,2]. The slotted waveguide antenna, when applied in phased array systems, has significant advantages in complex electromagnetic environments, and it can achieve many beneficial characteristics, such as fast beam tracking, beamforming, etc. [3,4,5,6]. However, to date, there has been limited research attention directed toward the circularly polarized phased slotted waveguide antenna. Based on our research (1), the dimensions of the waveguide and polarizer are the primary limiting factors regarding the beam scanning range of the phased slotted waveguide antenna, as these large structures result in a notably narrow scanning range. Consequently, this article introduces a novel approach: a compact circularly polarized non-resonant slotted waveguide, aimed at achieving a broader scanning angle.

Typically, a phased slotted waveguide array performs the phase dimensional beam scanning along the waveguide arrangement direction. This array commonly operates under resonant conditions, radiating linearly polarized electromagnetic waves [7,8,9,10,11]. The radiation elements typically consist of a series of slots positioned along the narrow wall of the waveguide. According to the basic phased array theory, the maximum achievable phase scanning angle satisfies the following:

(1) $$\theta_{max}=arcsin\left(\frac{\lambda }{d}-1\right)$$

where *d* is the element distance. In the slotted waveguide array, *d* represents the distance between adjacent linear waveguides. The grating lobe will manifest in the visual space if the scanning angle exceeds $\theta_{max}$.

**Figure 1:**
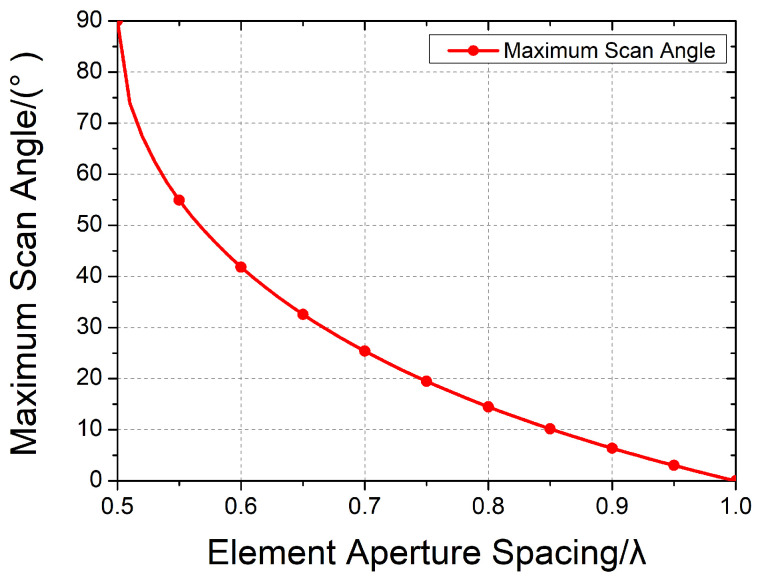
The relationship curve between the element aperture spacing and maximum scan angle.

Due to the limitation of the element distance, it is challenging to design a phased scanning slotted waveguide array with multiple polarizations. However, the regular fixed-beam slotted waveguide antenna arrays offer numerous valuable electric characteristics, such as wide-band polarization [12], dual-band, dual polarization [13,14,15], and circular polarization. Circularly polarized slotted waveguide arrays can be categorized into three distinct types based on their circular polarizer structures: the compound slots, the circular polarization patch or dipole, and the circular polarization (CP) cavity. Compound slots typically consist of a pair of slots [16,17] or some complex slots [18] that are carved into the wall of the waveguide. This type of CP element has the lowest profile and simplest configuration. However, achieving identical phase excitation requires the compound slots to be spaced at $\lambda_{g}$, necessitating the use of dielectric-filled waveguides or SIW to reduce the waveguide’s wavelength [19]. While dielectrics offer advantages in miniaturization, they also introduce additional electromagnetic attenuation losses, and many dielectric materials can compromise structural integrity. Another option is the ridge gap waveguide [20], but it also faces difficulties in miniaturization. The CP patch, dipole, and CP cavity are loaded on the waveguide slots, serving as polarizers. The CP patch or dipole has a flexible configuration, offering an excellent circular polarization performance (e.g., high efficiency [21], broadband CP [22]), and patches are easily combinable with metamaterials and other novel technologies [23,24,25]. However, the compact patches and dipoles loaded on the waveguides need dielectric layers, and compactly arranged patches may induce more mutual coupling effects, limiting the power capacity. On the other hand, CP cavities are typically open-ended waveguides with specific configurations [26,27]. The cavity aperture can significantly reduce the mutual coupling between elements and enhance the isolation performance, being particularly beneficial in non-resonant and phased arrays with complex aperture distributions. Cavities also offer greater robustness compared to patches. Nevertheless, cavities pose challenges in miniaturization: the conventional CP cavity’s aperture typically exceeds 0.6λ, according to Equation (1); this dimension limits the widest scanning angle to ±40°. Figure 1 clearly illustrates these limitations. The study in [28] introduced a prospective approach to miniaturization employing a four-ridged cavity and a center-set slot to reduce the aperture of the element. However, this study lacked comprehensive details regarding the antenna and primarily concentrated on a small resonant array. Drawing from the insights of ref. [28], we analyzed its construction and operational principles, expanding its applications to non-resonant arrays and phased arrays. Meanwhile, we addressed several challenges in integrating this compact structure into complex arrays. Additionally, the cavity’s CP frequency band is narrower than the bands of some CP patches [29].

The miniaturization of the waveguide slots poses another challenge in phased array antennas. As discussed above, the broad wall slots in phased arrays result in a very limited scanning angle. While reducing the width of the waveguide using ridge waveguides [8,9] is an option, designing them with circular polarizers is not easy. The asymmetric ridge waveguide presents a potential solution [30]; however, existing analyses have primarily focused on resonant arrays. To our knowledge, there is a dearth of research addressing phase scanning slotted waveguide arrays generating circularly polarized waves. The non-resonant slotted waveguide array offers a low-cost solution for two-dimensional beam scan arrays, integrating phase and frequency scanning within the same array. This is particularly suitable for applications insensitive to frequency variations, such as meteorological radar. Excited by traveling waves, the non-resonant slotted waveguide has the capabilities for frequency scanning [31]. The frequency scanning direction is along the waveguide. With this capability, the non-resonant slotted waveguide array can offer a two-dimensional beam scan in both the phase domain and frequency domain [32]. Furthermore, compared to resonant arrays, the non-resonant array can accommodate a wider waveguide length range, enabling a wider bandwidth.

To address the aforementioned problem, this article proposes an improved antenna structure aimed at achieving a wide beam scanning angle and integrating frequency domain and phase domain scanning array antennas. Our work introduced improved waveguide feeding slots, a circular polarizer, and staggered array design methods; a phase scanning angle of ±45° and a frequency domain scanning range of +11° to +13° were achieved in this study.

The key contributions of this study are as follows:

1. A center-set waveguide slot element on the centerline of the broad wall is proposed to set up a waveguide circular polarizer with four ridges;
2. A compact circular polarized slotted waveguide planar array is presented that can realize a wide phase scanning range.

The rest of this article is organized as follows. Section 2 introduces the design of the compact antenna element, including the circular polarizer and the center-set waveguide slot. Section 3 introduces the slotted waveguide array designed using this compact circularly polarized element, along with the technical details concerning beam scanning in the array design. Section 4 presents the simulation and the measured results. Finally, the conclusions are presented in Section 5.

## 2. Configuration of the Antenna Element

**Figure 2:**
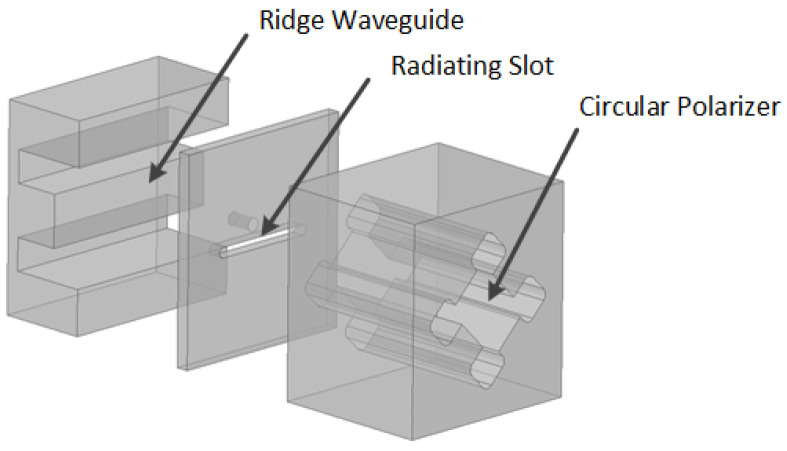
Configuration of the proposed antenna element.

The proposed slotted antenna element configuration is depicted in Figure 2. Comprising two components, namely, the circular polarizer and the slotted ridge waveguide, the element functions as follows. A single-ridge waveguide with a straight slot positioned along the centerline of the broad wall serves as a feed waveguide. A metallic cylinder placed near the slot disturbs the electromagnetic field distribution within the waveguide. The slotted ridge waveguide emits linearly polarized waves through the straight slot on the waveguide. The circular polarizer, configured as an open-ended quad-ridge waveguide, is mounted on the slot at a $45^{\circ }$ angle.

### 2.1. Design of Circular Polarizer

The circular polarizer is implemented using an open-ended four-ridge waveguide. Excited by the slot, the circular polarizer reflects linearly polarized waves fully from the cavity walls, generating two orthogonal electric field components: the *TE*_10_ mode and the *TE*_01_ mode. In properly adjusting the polarizer’s profile, an equal amplitude and a $90^{\circ }$ phase gradient between these two propagating modes can be achieved. Consequently, upon reaching the radiating aperture interface, the combination of these two modes yields circularly polarized waves. The primary objective of the waveguide circular polarizer is to propagate the *TE*_10_ and *TE*_01_ modes while suppressing the *TE*_11_ mode within the operating frequency band.

**Figure 3:**
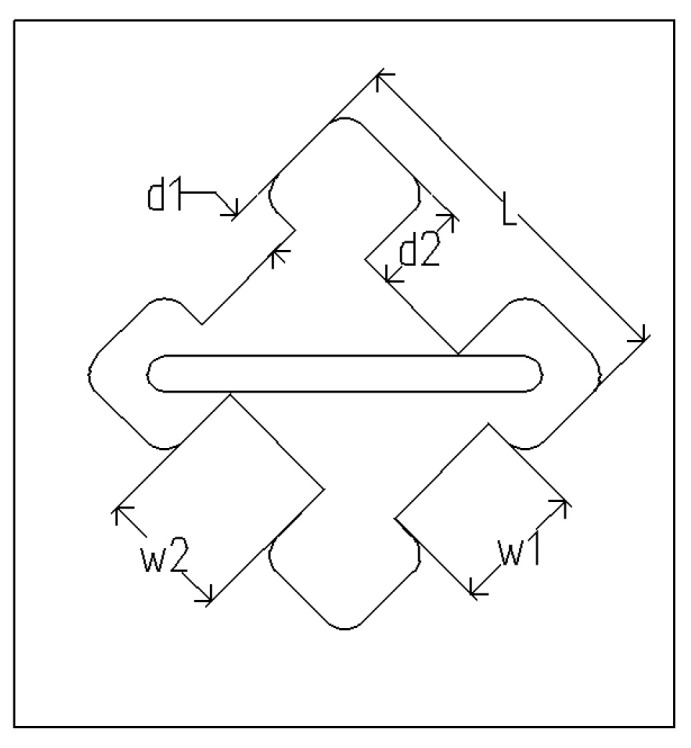
Top view of the circular polarizer.

**Figure 4:**
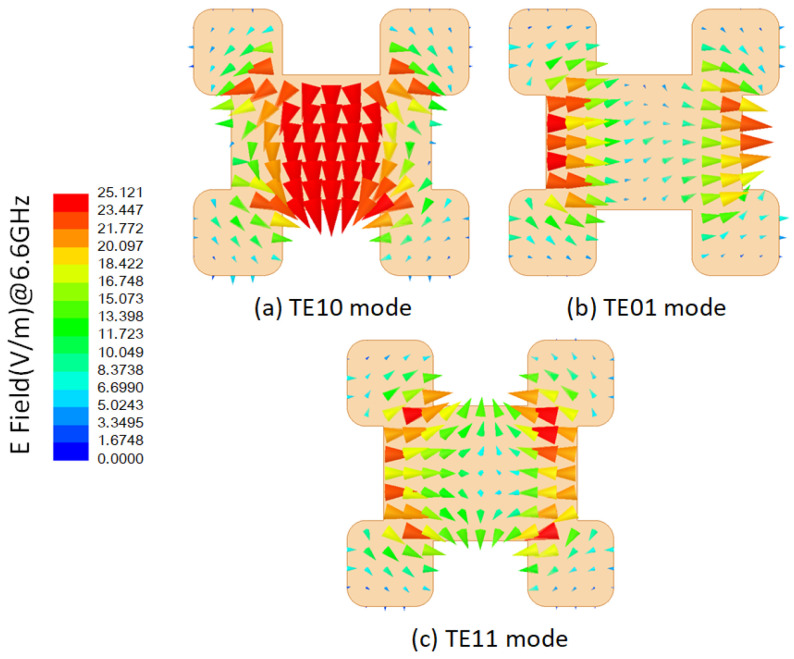
Electric field distributions of the three modes in a circular polarization cavity at 6.6 GHz.

A cross-section of the circular polarizer is depicted in Figure 3. The outer shape of the polarizer is a square, with four ridges positioned inside, where the opposite ridges are of equal size. The parameters of the ridges significantly impact the propagation characteristics of the electromagnetic wave modes. Specifically, the lower ridges primarily affect the *TE*_10_ mode, while the higher ridges influence the *TE*_01_ mode. Moreover, due to their close proximity, the *TE*_11_ mode is susceptible to influence from both the *TE*_01_ and *TE*_10_ modes. The electric field distributions of these three modes in the polarizer cross-section are shown in Figure 4.

**Figure 5:**
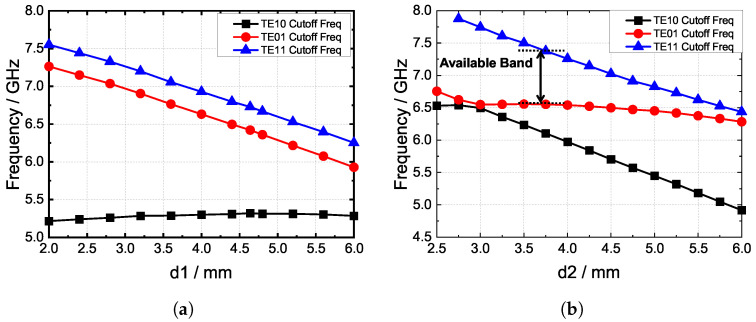
(**a**) The relationship between the cutoff frequencies and ridge height d1 and (**b**) the relationship between the cutoff frequencies and ridge height d2.

The parameter values are subject to several conditions. The outer size of the polarizer is determined by the dimensions of the element radiator, which, ideally, should be minimized. The width of the ridges is contingent upon the fabrication accuracy requirements and had a negligible influence on the cutoff frequencies in this study. Consequently, the analysis focused on the ridge parameters to ascertain specific changes in the cutoff frequency. As depicted in Figure 5, d1 and d2 exert significant effects on the cutoff frequencies of the three modes. The simulated results align with the prior analysis; Figure 5a illustrates that d1 can alter the cutoff frequencies of the *TE*_10_ and *TE*_11_ modes, with their curves exhibiting similar trends, whereas the performance of the *TE*_01_ mode is minimally affected by changes in d1. Similarly, Figure 5b demonstrates that variations in d2 have a similar effect than those in d1, albeit with an emphasis on the *TE*_10_ and *TE*_11_ modes. Our objective is to preserve the *TE*_01_ and *TE*_11_ modes while suppressing the *TE*_11_ mode within the operating frequency band. The available bandwidth is indicated by the black arrow in Figure 5b. By finely adjusting these parameters, we can effectively control the propagating modes of the polarizer.

The cutoff frequencies of each mode, as well as the waveguide wavelength, are obtained according to the parameters of the ridges. In order to satisfy the phase difference between the two modes, the height of the polarizer satisfies the following:

(2) $$\frac{2\pi h}{\lambda_{g10}}-\frac{2\pi h}{\lambda_{g01}}=\frac{\pi }{4}$$

where $\lambda_{g10}$ is the waveguide wavelength of the *TE*_10_ mode, $\lambda_{g01}$ refers to the waveguide wavelength of the *TE*_01_ mode, and h is the height of the polarizer.

With the aforementioned analysis, the dimensional parameters of the polarizer are established. The accepted design parameters are listed in Table 1.

### 2.2. Slotted Waveguide with Metallic Cylinder Inside

**Figure 6:**
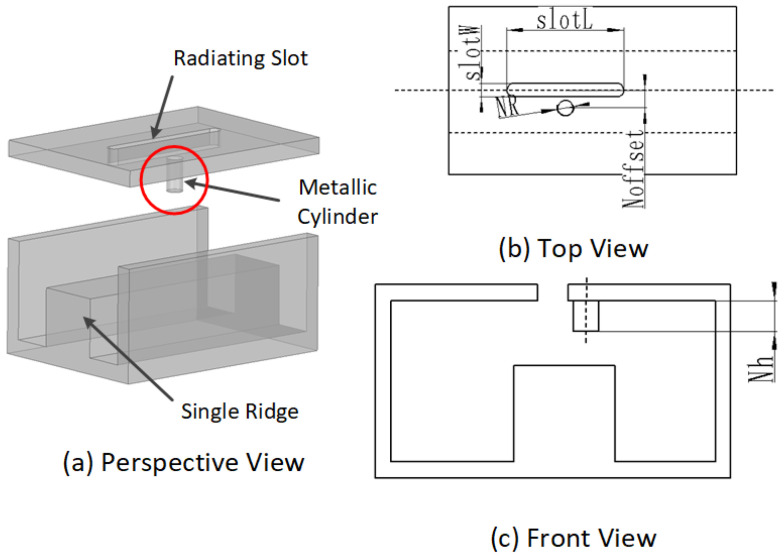
The configuration of the proposed feed-slotted waveguide. (**a**) Perspective view. (**b**) Top view. (**c**) Front view.

A linear center-set slotted waveguide array with a compact ridge is proposed. Longitudinal slots, serving as radiating elements, are positioned along the centerline of the waveguide’s broad wall. Metallic cylinders are staggered on the upper inner wall of the waveguide, on alternating sides of the slots. The configuration of the single-slot radiator is illustrated in Figure 6.

Conventional waveguide slots are typically carved at specific offset distances from the centerline of the broad wall. However, as previously discussed, the polarizer is loaded above the center of each slot at a $45^{\circ }$ rotated angle. Consequently, conventional offset slots would lead to offset polarizers, resulting in wider antenna elements. With the proposed arrangement of the waveguide slots and polarizers, the element width can be significantly reduced. This approach allows all radiating slot elements to be arranged in a regular grid, eliminating the need for offsetting them as in traditional designs.

**Figure 7:**
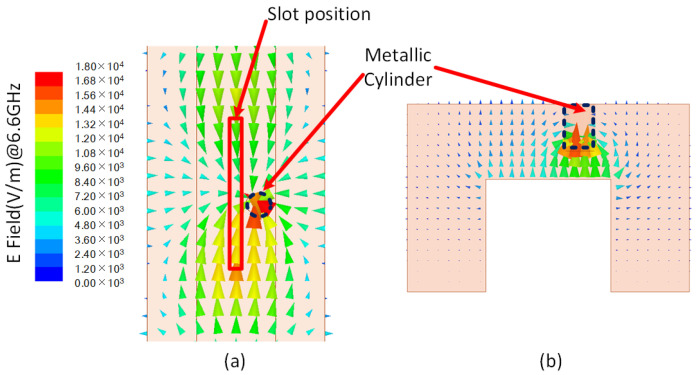
The field distribution of the waveguide with a cylinder. (**a**) The electric field in the upper wall of the waveguide. (**b**) The electric field in the cross-section of the waveguide.

The introduction of special structures into the waveguide can alter its electromagnetic performance [33,34]. As depicted in Figure 6, a metallic cylinder is positioned on the inner upper wall of the waveguide at a specific offset distance from the centerline. The role of the cylinder is to disrupt the electromagnetic field distribution within the waveguide, resulting in a concentration of the electric field. In the conventional ridge waveguide, the waveguide’s upper wall and the ridge’s upper wall can be approximated to a plate capacitor. The capacitor’s field distribution and voltage performance are influenced by the two plates’ distance, which is the distance between the waveguide upper wall and the ridge upper wall in this situation. With a metallic cylinder introduced into the waveguide, the capacitor model is altered to three capacitors; the cylinder’s bottom surface and the ridge’s upper surface are approximated to a capacitor, and the remaining parts are approximated to other capacitors. According to capacitor theory, the closer plates have a higher voltage. Hence, the voltage at the cylinder is higher than that in other areas. Figure 7 illustrates the field distribution of the waveguide with the cylinder, showing the gathering of electric field and surface current at the cylinder position. This disruption leads to an asymmetric distribution of surface current, causing an electric potential difference at the centerline of the waveguide. Under these conditions, the center-set slot can be effectively excited by the asymmetric surface current, with the available slot positions indicated by red marks in Figure 7a. Through the preceding analysis, it is evident that both the location and scale of the cylinder influence the field distribution.

**Figure 8:**
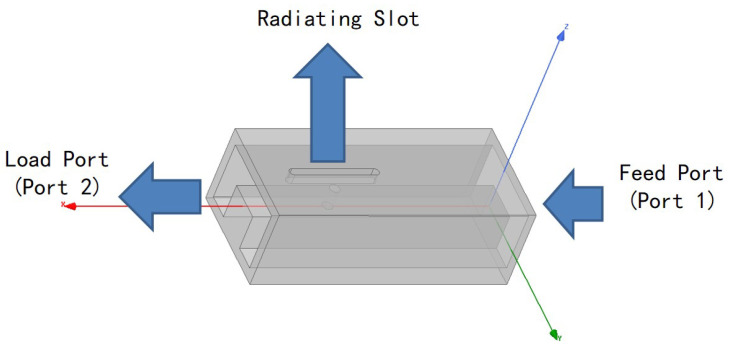
The simulation model of the slotted waveguide element.

**Figure 9:**
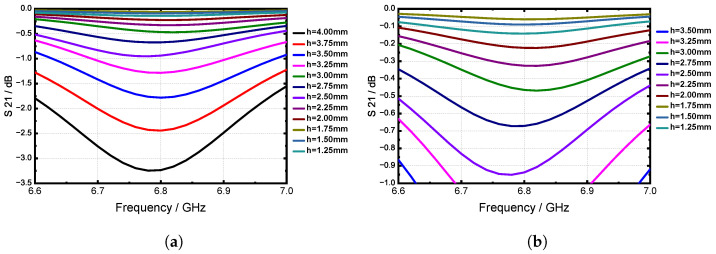
(**a**) The simulated S21 (dB) curves of the single resonant slot when changing the cylinder height and (**b**) the enlarged view for h = 2.50 mm to 4.00 mm.

Basic slotted waveguide antenna theory indicates that the radiation efficiency is contingent upon the excitation voltage of the slot. Leveraging these theories, we adjusted the parameters of the cylinder to analyze the impact on the radiation performance of the waveguide slot. Figure 8 depicts the simulation model of the slotted waveguide element. Figure 9 depicts the S21 results for various cylinder heights under resonant conditions. The simulation results demonstrate that longer cylinders result in more radiated energy. Furthermore, while the offset distance of the cylinder does influence radiation efficiency, its effect is not particularly significant; rather, it primarily affects the gathering position of the surface current. The radius of the cylinder impacts the intensity of the reflected wave, with thicker cylinders potentially being intersected by the slot, resulting in irregular shapes and complex field distributions. Additionally, the slot length can be adjusted to tune the resonant frequency of the element, akin to conventional waveguide slots.

**Figure 10:**
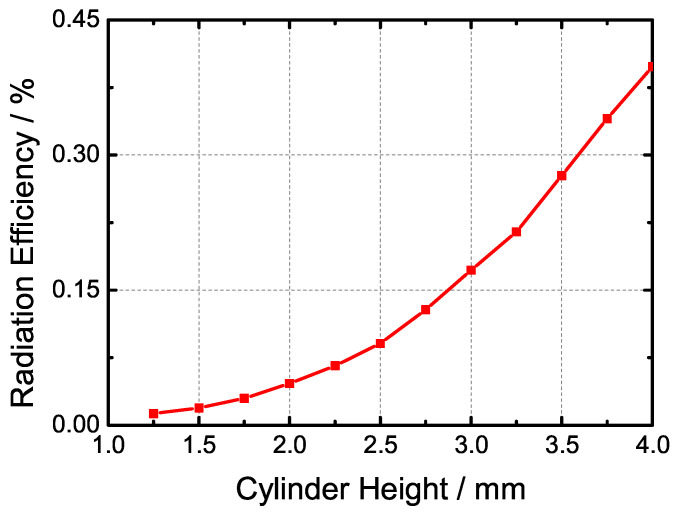
The relationship between the simulated radiation efficiency and the cylinder height under the resonant condition.

Following the above discussion, we can manipulate the radiation efficiency by adjusting the height of the cylinder. The relation between the radiation efficiency of the slot and the cylinder height is shown in Figure 10; the cylinder height refers to *Nh* in Figure 6c. the radiation efficiency is defined as follows:

(3) $$\;Eff_{rad}=1-{\left(S11\right)}^{2}-{\left(S21\right)}^{2}$$

where $\;Eff_{Rad}$ refers to the radiation efficiency of the antenna element, S11 is the energy reflected back to the excited port, and S21 is the energy transmitted to the load or next element.

A method for controlling the element’s radiation efficiency is introduced in this section; it lays the foundation for the follow-up array design work.

This section describes the working principle and design method of a compact circularly polarized antenna element, resulting in a miniaturized circularly polarized antenna element with an aperture size of 0.48λ × 0.48λ. Additionally, this section introduces the method used to control the radiation efficiency of the element, which serves as the foundation for subsequent design considerations.

## 3. Design of Antenna Array

### 3.1. Linear Array

**Figure 11:**
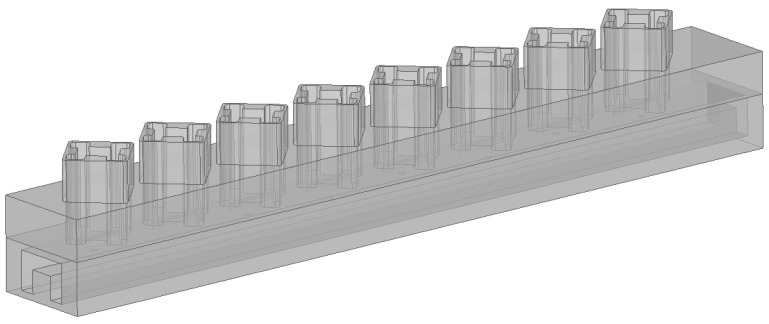
Configuration of an eight-element linear array.

In this section, the compact antenna element proposed in Section 2 is arranged into a linear array, with each antenna element featuring a center-set waveguide and a compact circular polarizer. They are fed by a straight-through waveguide tube, and the structure of this linear array is shown in Figure 11. The linear array is a non-resonant array that is excited by a traveling wave. In non-resonant arrays, the slots are excited with different phases, resulting in the main lobe beam deviating from the normal direction. The phase difference between the slots varies with the frequency, causing the main lobe direction to change accordingly. Thus, non-resonant arrays possess the capability of frequency scanning. In drawing on fundamental phased array antenna theory and waveguide theory, the relationship between the slots distance *d* and the beam angle θ satisfies the following:

(4) $$\;\theta =arcsin(\frac{\lambda }{\lambda_{g}}-\frac{\lambda }{2d})$$

The distance between the slots also impacts the resonant frequency of the linear array. When the slot distance is $0.5\lambda_{g}$, the non-resonant array exhibits a very high VSWR at the resonant frequency [35,36]. This phenomenon can be mitigated by increasing the slot distance. However, excessively long slot distances will result in a high grating lobe level. The suppression condition for grating lobes can also be described as in Equation (1). In conclusion, the maximum value of the slot distance is constrained by the performance of the grating lobe level, while the minimum value is determined by the VSWR performance. Additionally, the main lobe direction must be considered in the determination of the slot distance.

The control of the first side-lobe level was also achieved in this work. Side-lobe control in slotted waveguide arrays is accomplished by regulating the radiation power weighting of the elements. The weighting factor is determined using the Taylor Pattern Synthesis Method. In building upon the findings from Section 2, the differential power weighting of the elements is achieved through adjustments in the height of the cylinder and the length of the slot.

### 3.2. Planar Array

**Figure 12:**
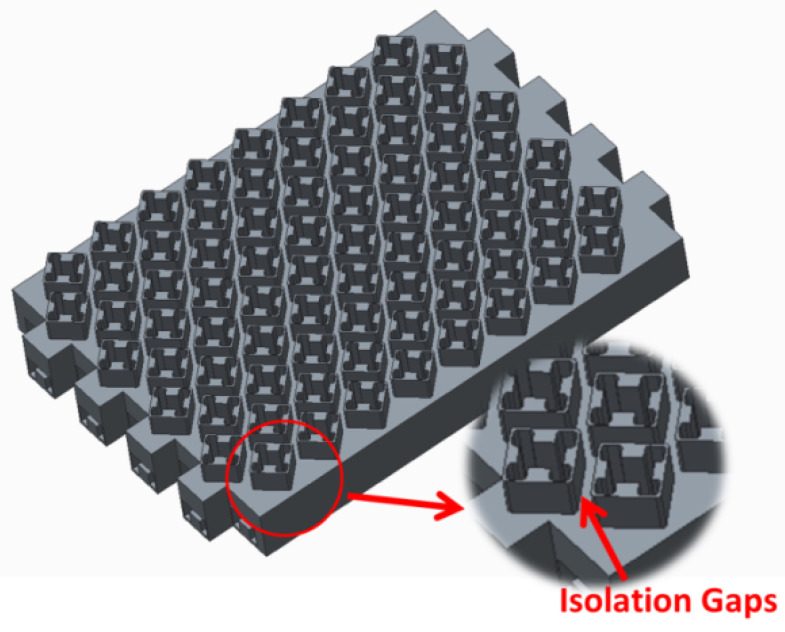
Configuration of the planar array.

**Figure 13:**
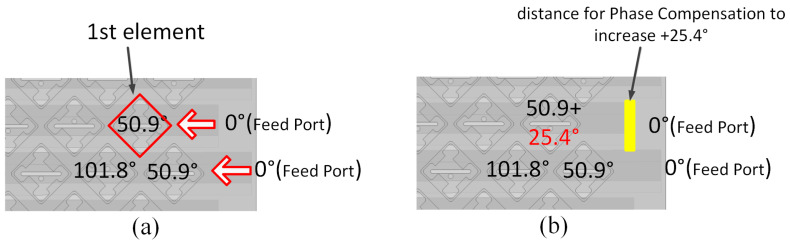
(**a**) The mismatching phase distribution in the planar array. (**b**) The compensation phase distribution in the planar array.

**Figure 14:**
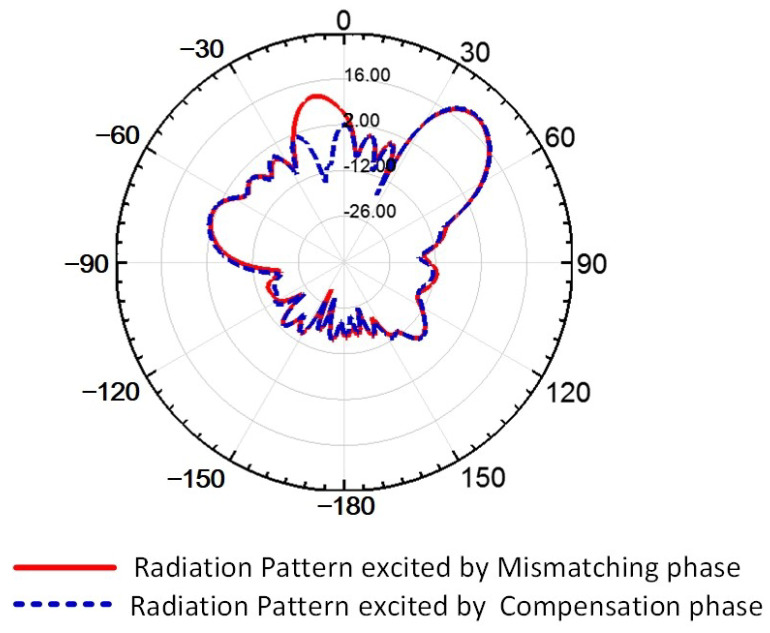
The red line depicts the simulated radiation pattern under the mismatching phase condition, and the blue line depicts the simulated radiation pattern under the compensation phase condition, at a 45° beam scanning angle through phase scanning.

In this section, the parallel waveguide linear array described in Section 3.1 is arranged into a two-dimensional array, and the structure of the planar array is shown in Figure 12. To reduce the width of the linear array, we adopted the staggered arrangement method to position adjacent linear arrays. However, this staggered arrangement results in an asymmetric planar array, leading to high axial ratio levels and elevated, far side-lobe levels. To address this issue, we staggered the placement of the eight-element and nine-element linear arrays. However, the staggered non-resonant array exhibits a less-than-optimal phase distribution. As depicted in Figure 13a, the phase distribution of the eight-element array does not align with that of the nine-element array. This mismatched phase distribution exacerbates the grating lobe performance during phase scanning, as indicated by the red line in Figure 14. To remedy this, we increase the excitation phase of the eight-element arrays to compensate for the phase offset induced by the staggered arrangement (Figure 13b). The magnitude of the increased phase is determined by the waveguide wavelength $\lambda_{g}$ and the slot distance *d*. The precise expression for the compensated phase is given by

(5) $$\;Phase_{comp}=\left(\frac{d}{\lambda }-\frac{1}{2}\right)\pi$$

As shown in Figure 13, the phase compensation can be achieved by increasing the distance between the excite port and the first element. Figure 13a shows the situation without phase compensation; the neighboring elements exhibit non-uniform phase excitation, and the red arrow indicates the direction of the electromagnetic wave transmission. In Figure 13b, we introduce an additional distance between the first element and the feed port, as highlighted by the yellow rectangular marker; subsequently, the first element’s working phase is compensated for to achieve the correct value. The compensated result is depicted by the blue line in Figure 14, and the grating lobe was suppressed effectively by applying this method.

**Figure 15:**
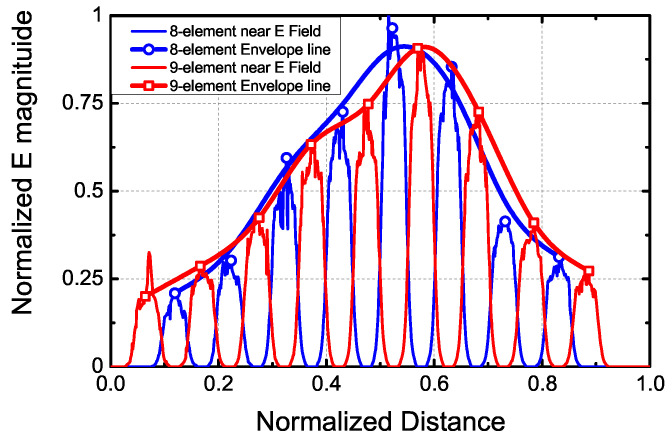
The near E-field curves and its envelope line of the adjacent eight- and nine-element linear arrays.

With the introduction of the nine-element array into the planar array, the power weighting of the elements requires re-tuning. Similar to phase compensation, the radiation fields of the eight-element and nine-element arrays also need to be aligned. To address this, we utilize the near field results, as depicted in Figure 15. The radiation performance of the eight-element array serves as the reference standard. By plotting the envelope line of the near radiated electric field, we can approximate the changing curve of the near field for the eight-element linear array. The design parameters of the nine-element array are adjusted to match the curve extracted from the eight-element array. Initially, the weighting parameters of the nine-element array are defined using the pattern synthesis method, and the initial near field curve is extracted and compared with that of the eight-element array. Based on the discrepancy between the two curves, adjustments are made to the cylinder height and slot length of the nine-element array to ensure that its curve matches the standard curve. This iterative process is repeated several times throughout the design phase, resulting in the final curves depicted in Figure 15. The results demonstrate the good alignment between the two curves.

As discussed earlier, the staggered arrangement results in a complex near field distribution, exacerbated by the excitation of traveling waves, leading to the intricate phase distribution of the near field. Additionally, the compact arrangement of the radiating cavities exacerbates the coupling effects compared to normal conditions. In such scenarios, the planar array’s leakage wave on the radiating surface exhibits a complex phase and field distribution, adversely affecting the axial ratio level. To mitigate the influence of the leakage wave, isolation gaps are introduced on the radiating surface. These gaps serve as channels into which the leakage wave can be transmitted, subsequently being reflected at the bottom of the gaps. By adjusting the depth of the gap, the reflected wave and transmitted wave can be neutralized within it. The configuration of the isolation gaps is illustrated in Figure 12.

With the above analyses and designs, we can obtain the final configuration of the planar array antenna. The detailed parameters are presented in Table 2.

## 4. Verification and Comparison

**Figure 16:**
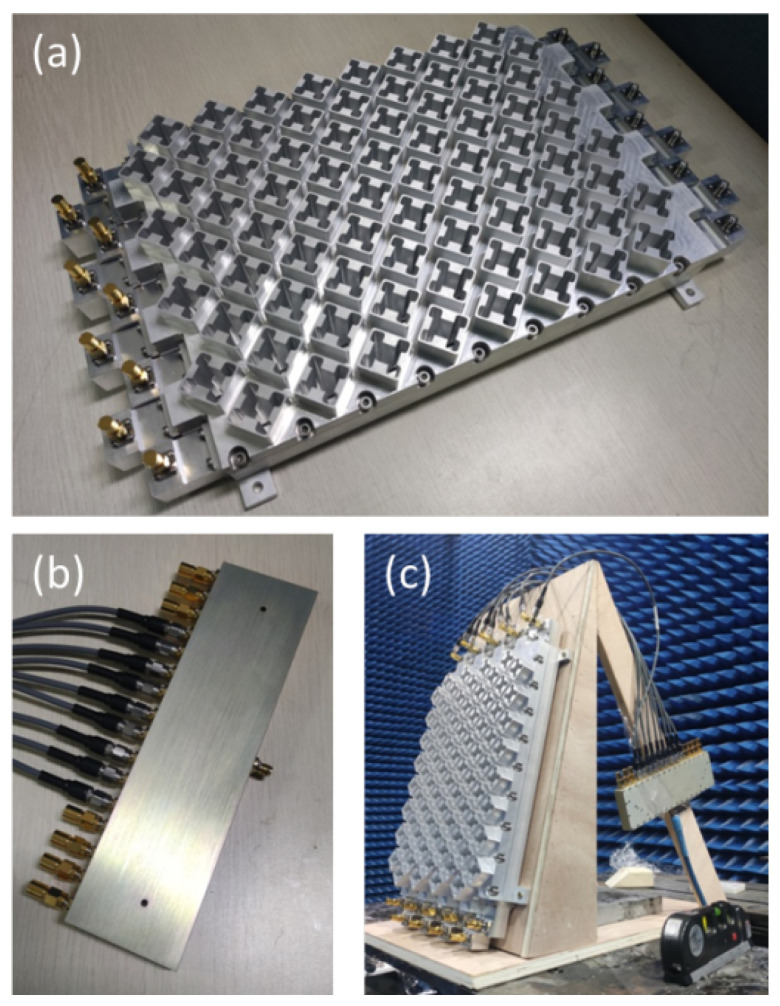
(**a**) The fabricated antenna. (**b**) The power divider. (**c**) The experimental setup for measurements.

The planar antenna was fabricated and measured in our work. During manufacturing, the antenna is divided into three layers: the waveguide layer, including the waveguide and ridge structure; the slot layer, including the slots and cylinders; and the circular polarizer layer, including the circular polarizer and isolation gap surface. The planar antenna was manufactured using CNC machining technology. During assembly, a conductive adhesive was applied to bond the waveguide layer and slot layer. This prevents the deterioration in the electrical performance caused by electromagnetic leakage resulting from the interlayer gaps; the circular polarizer layer and waveguide layer were mechanically connected using screws, which were distributed around the periphery of the prototype. The mechanical strength is the primary consideration when using screw connections, as the three layers are heavy and cannot withstand the required strength solely through adhesive bonding. The measurement was conducted using the far-field method in a 9 m × 9 m microwave anechoic chamber. The assembled antenna is depicted in Figure 16a, with dimensions of 370 mm × 240 mm × 52 mm.

The excitation method warrants mention. Although this antenna is intended for phased scan array applications, constructing a complete phased array system for measurement purposes is impractical. Instead, in this study, the phase delay was achieved using nine coaxial cables of varying lengths, ensuring approximate phase differences of 144 degrees. These cables were connected to a power divider and the antenna, as depicted in Figure 16b. The experimental setup is illustrated in Figure 16c, showing a 12° inclined support being employed to accommodate the deviated main lobe in the elevation plane.

### 4.1. Simulation and Measurement Results

**Figure 17:**
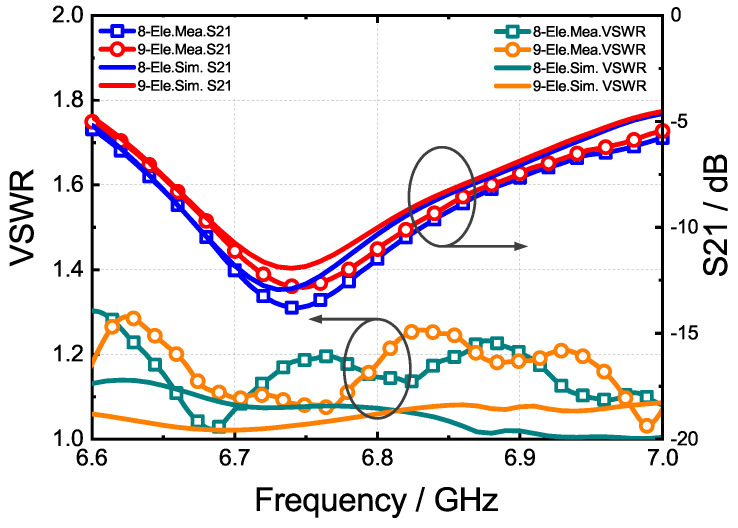
The simulated and measured VSWR and S21 curves within the work band.

The prototype antenna was verified, and the simulation and measurement results are presented below. The VSWR and S21 of the eight- and nine- element linear arrays were measured, respectively, abd the results are shown in Figure 17. It is observed that the measured results closely align with the simulation, indicating good agreement.

**Figure 18:**
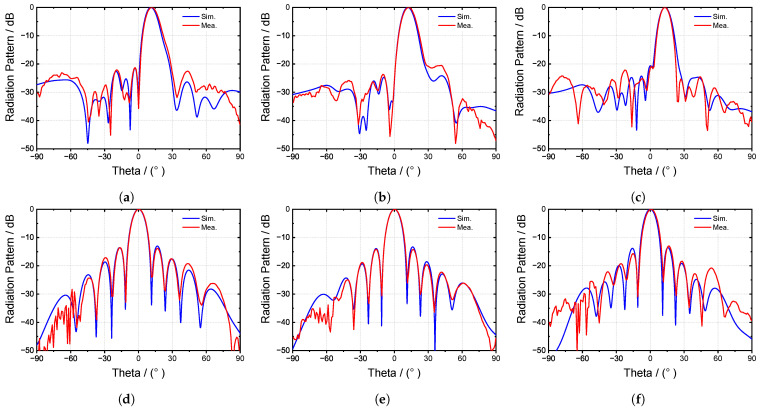
The measured and simulated radiation patterns in the elevation (EL.) and azimuth (Az.) planes. (**a**) El. plane at 6.6 GHz. (**b**) El. plane at 6.8 GHz. (**c**) El. plane at 7.0 GHz. (**d**) Az. plane at 6.6 GHz. (**e**) Az. plane at 6.8 GHz. (**f**) Az. plane at 7.0 GHz.

Figure 18 presents the radiation patterns of the proposed antenna. Figure 18a–c depict the elevation radiation patterns at 6.6 GHz, 6.8 GHz, and 7.0 GHz, respectively. The majority of the simulation and measurement results exhibit close agreement. However, there are slight discrepancies in the right side-lobe level (SLL) at 6.8 GHz, although all measured SLL results remained below −20 dB. Furthermore, the frequency scan angle results align well with those of the simulation. The azimuth radiation pattern results are depicted in Figure 16d–f; all measured azimuth radiation results exhibit good concordance with the simulation data. Additionally, the achieved side-lobe levels (SLLs), at approximately −14 dB, are attributed to the utilization of an equal-amplitude power divider.

**Figure 19:**
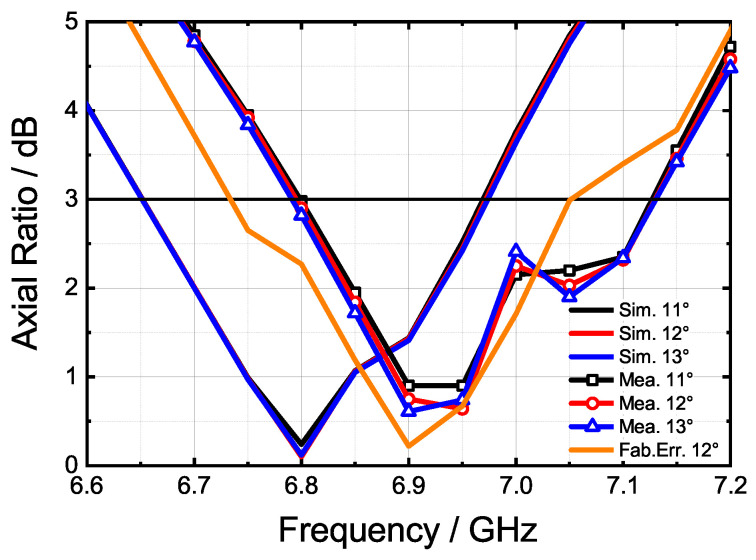
The measured and simulated axial ratios at 11°, 12°, and 13° at 6.6–7.2 GHz.

The axial ratio results are shown in Figure 19 and Table 3. A slight offset of 100–150 MHz is observed in the measured results compared to the simulation results, although the axial ratio frequency bands remain consistent. After the measurement, it was determined that the error in these results primarily arose from two sources: manufacturing and assembly errors. Specifically, the measured value of parameter *d*1 (in Section 2) ranges between 2.9 and 3.1 mm, and the depth of the circular polarizer varies within 31.4 and 31.6 mm, both of which deviate from the intended design values. It was found that the conductive adhesive method of the waveguide cavity layer and gap layer does not affect the electromagnetic performance. However, the screw connection between the circular polarizer layer and the waveguide layer has an impact on the axial ratio. This is due to the deformation of the slot layer and the circular polarizer layer at the center of the prototype. The simulation results, considering the fabrication error, are indicated in Figure 19 by the orange line. To address this problem, the circular polarizer layer and the waveguide layer should be connected using both screws and conductive adhesive, and the position of the screws needs to be redesigned to address the deformation problem after assembly. Enhancing the fabrication accuracy and applying an adhesive to the metal layers can mitigate the frequency offset.

**Figure 20:**
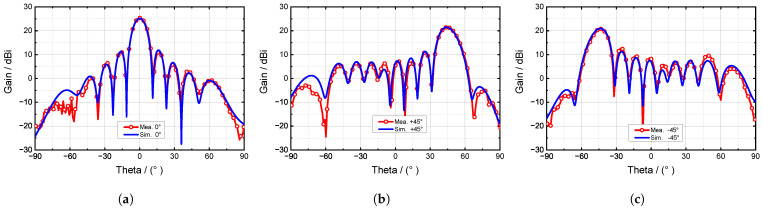
The measured and simulated phased scan radiation patterns in the azimuth plane: (**a**) in the normal beam direction; (**b**) in the +45° beam direction; (**c**) in the −45° beam direction.

The advantage of the proposed antenna is the wide beam scan angle; the phase scan radiation pattern in 6.8 GHz is plotted in Figure 20. The results show that the antenna can realize beam scanning in the range of −45°∼+45° without a grating lobe; the phase mismatching effect is entirely suppressed. Table 4 shows the axial ratio result in the phased scan condition; the scanned beam can still maintain a 3 dB axial ratio under the machine error.

### 4.2. Comparison and Discussion

Table 5 compares the performance of the proposed antenna with those of several circularly polarized waveguide slot antennas reported in the literature. Our work achieved a beam scanning range of −45°∼+45° in the azimuth plane and a scanning range of +11°∼+13° in the elevation plane. Refs. [19,20] pertain to frequency scan arrays, while [22] utilized a wideband CP dipole to expand the axial ratio (AR) band. Ref. [28] employed a similar structure antenna element and proposed a fixed beam array; however, the authors did not focus on the compact array research domain. Notably, our work achieved a low side-lobe level (SLL). Comparatively, our antenna realizes a larger beam scanning range while maintaining an average or superior electrical performance in other aspects.

## 5. Conclusions

This paper proposes a compact circularly polarized non-resonant slotted waveguide array antenna with a wide-beam scanning angle ability. We modified and improved the existing compact slotted waveguide antenna through a thorough analysis of its working principles and the optimization of design parameters, adding isolation gaps, and adapting it for use in non-resonant arrays and phased arrays. The array’s performance was optimized through phase compensation and element power matching techniques. As a result, we achieved a compact slotted waveguide antenna that can work in non-resonant arrays with the capability for wide beam scanning angles. The prototype antenna is proposed, and the experimental results prove the effectiveness of the antenna design methodology. The designed antenna exhibits a phase scan capability of ±45° in the azimuth plane and a frequency scan range of +11° to +13° in the elevation plane, without the grating lobe, and it maintains a low SLL. The axial ratio remains below 3dB within this scanning range and bandwidth. Overall, the proposed antenna effectively combines circular polarization with wide-angle beam scanning capabilities in slotted waveguide array antennas.

## Figures and Tables

**Table 1:** The parameter values of the four-ridge cavity design.

Parameters	s1	s2	d1	d2	h
Value (mm)	7.5	7.5	2.9	5.3	31.3

**Table 2:** Detailed parameters of the planar array (unit: mm).

Slot No.	Eight-Element Array’s Cylinder Height	Eight-Element Array’s Slot Length	Nine-Element Array’s Cylinder Height	Nine-Element Array’s Slot Length
1	1.7	21.6	1.6	21.6
2	2.2	21.4	1.9	21.4
3	2.8	21.4	2.5	21.4
4	3.3	21.4	3.1	21.4
5	3.9	21.1	3.4	21.1
6	4.1	20.9	3.9	20.9
7	3.8	20.9	4.0	20.9
8	3.2	21.1	3.5	21.1
9	N/A	N/A	3.1	21.4

N/A = Not Applicable.

**Table 3:** The simulated axial ratio (AR) results in the azimuth plane.

Frequency/GHz	AR at 11°/dB	AR at 12°/dB	AR at 13°/dB
6.7	2.01	2.01	2.00
6.8	0.24	0.10	0.12
6.9	1.44	1.43	1.41
7.0	3.76	3.71	3.64

**Table 4:** A comparison of the measured (Mea.) and simulated (Sim.) axial ratio results for a 6.8 GHz phase scan.

Scan Angle	−45°	0°	+45°
Axial Ratio (Sim.)	3.33 dB	0.23 dB	2.69 dB
Axial Ratio (Mea.)	2.3 dB	2.9 dB	2.2 dB

**Table 5:** Performance of the waveguide slot planar array.

Ref.	Feed Type	Polarizer Type	Number of Elements	Excitation Wave Type	Frequency Band (%)	El. Plane SLL (dB)	Gain (dBi)	Axial Ratio Band (%)	Scan Type	Scan Angle (°)
[17]	Waveguide	Slots	1 × 15	Traveling	1.33	−18 (Sim.)	21 (Sim.)	>2.7 (Sim.)	-	-
[19]	SIW	Slots	64 × 12	Traveling	8.03	−14	28	-	Freq	+36∼+49 (El.)
[20]	RGW	Slots	1 × 7	Traveling	21.43	-	12.5	21	Freq	−15∼+15 (El.)
[21]	Waveguide	Patches	1 × 10	Standing	2.85	−13	15.94	3.2	-	-
[22]	Waveguide	Dipoles	12 × 12	Standing	7.33	−12	28.01	14 (Sim.)	-	-
[28]	Waveguide	Waveguide	2 × 8	Standing	1.63	−13 (Sim.)	17 (Sim.)	-	Phase	-18∼+18 (Az.)
This work	Waveguide	Waveguide	5 × 8& 4 × 9	Traveling	5.89	<−20	24.3	5.0	Freq and Phase	+11∼+13 (El.) −45∼+45 (Az.)

SIW = substrate integrated waveguide; RGW = ridged gap waveguide; Sim. = simulation result; El. = beam scan in elevation plane; Az. = beam scan in azimuth plane.

## Data Availability

The data presented in this study are available from the corresponding author upon request.
